# Influence of Peanut Varieties on the Sensory Quality of Peanut Butter

**DOI:** 10.3390/foods11213499

**Published:** 2022-11-03

**Authors:** Tapiwa Reward Sithole, Yu-Xiang Ma, Zhao Qin, Hua-Min Liu, Xue-De Wang

**Affiliations:** College of Food Science and Engineering, Henan University of Technology, Zhengzhou 450001, China

**Keywords:** peanut varieties, peanut butter, peanut butter sensory quality

## Abstract

Over the years, concentrated efforts have been directed toward the improvement of desirable characteristics and attributes in peanut cultivars. Most of these breed improvement programs have been targeting attributes that involve peanut growth, productivity, drought and disease tolerance, and oil quality and content, with only a few articles focusing directly on improvements in peanut butter organoleptic qualities. There are numerous peanut cultivars on the market today, with widely differing chemical compositions and metabolite profiles, about which little is known concerning their suitability for making peanut butter. In this review, we detail how the numerous peanut varieties on the market today, with their genetically conferred physiochemical attributes, can significantly affect the sensory quality attributes of peanut butter, even in peanut butter processing lines with optimized processes. If other peanut butter processing parameters are held constant, variations in the chemical composition and metabolite profiles of peanuts have a significant impact on peanut butter color, flavor, texture, storage stability, shelf life, and overall product acceptance by consumers. Further research on breeding programs for peanut varieties that are specifically tailored for peanut butter production, and even more comprehensive research on the synergetic relationship between peanut chemical composition and peanut butter organoleptic quality, are still required.

## 1. Introduction

The production and consumption of peanut butter date back to over a century ago, starting with the ancient Incas and Aztecs, and they have greatly increased in recent years [1,2,3]. Growth in peanut butter consumption is primarily driven by the ever-increasing consumer demand for healthier food products, the growth in demand for convenient food, changing preferences, new tastes, and innovations in the food and beverages industries [3,4,5]. Furthermore, peanut butter is gaining more popularity, in particular as a good protein source that is ideal for individuals following a vegan or vegetarian diet and those people who are on a ketogenic diet (i.e., a high-fat adequate-protein, low-carbohydrate diet), such as epilepsy patients and athletes [5,6].

With the growth in the peanut butter market, there are now strong specific product expectations and preferences from consumers with regard to color, consistency, flavor, and perceived naturalness [7,8,9,10,11]. Apart from processing conditions, a clear and concise understanding of how peanut varieties and their respective specific chemical compositions and metabolite profiles influence organoleptic quality is essential to ensuring that peanut butter producers and peanut breeders are in sync with their customers’ quality expectations and that their customers are consistently satisfied. Given the increasing influence of online food reviews [12,13], even seemingly menial deviations from the peanut butter quality expectations of consumers might cause significant damage to the reputation of a peanut butter producer. In addition, with some apparent erosion of trust in food production companies, customers now tend to be more deliberate in their purchase choices, exercising more frugality than ever [14], becoming less tolerant of any variations to their quality expectations, and being more critical regarding food additives [13]. While the addition of some peanut butter additives, such as hydrogenated oils stabilizers, sugar, molasses, and salt, has for some time boosted peanut butter organoleptic appeal [9], nowadays, due to increasing health consciousness, some consumers are beginning to shun foods that contain additives [3,10,15,16,17]. For peanut butter, and indeed for many other food products, the mantra “the less the ingredients, the better” is becoming increasingly popular [15,18]. This might imply that, in the future, the organoleptic appeal of peanut butter will be driven mainly by the naturalness of the taste of the peanuts used and less reliant on additives. Accordingly, an inquiry into which peanut cultivars inherently possess superior physicochemical attributes for the production of a natural peanut butter (with less or no additives) that resonates well with consumers’ sensory quality expectations is increasingly a worthwhile endeavor.

While peanut butter color and flavor are upfront in generating initial consumer appeal and purchasing intention [19], a violation of consumers’ textural expectations can be hardly forgiven [20]. These sensory quality attributes of peanut butter (flavor, color, and texture) are primarily driven by the production processes (growth, handling, storage, roasting, grinding, and stabilization) [21], as well as by the intrinsic physical attributes and chemical compositions of specific peanut cultivars and additives [19,22]. Numerous efforts have been directed at gaining a mastery in optimizing the peanut butter production processes to achieve superior sensory quality [23,24,25,26,27]. Nevertheless, even with these optimized processes, if no due consideration is given to the variety of the peanuts that are processed, sensory quality variations of peanut butter might still be experienced, due to significant variations in the intrinsic physical and chemical compositions of different cultivars [22]. By extension, these quality variations that emanate from the differences in sensory intensities associated with specific peanut genotypes may influence consumer acceptance [28]. Therefore, it is important to understand how variations in the chemical composition and the metabolite profiles of peanuts affect the key quality attributes of peanut butter. This paper discusses the extent to which variations in peanut varieties and cultivars may impact the quality attributes of peanut butter and, subsequently, consumer acceptance. Thus, this paper provides a basis for further research in peanut breeding that is specifically tailored for making peanut butter. Furthermore, this information can be valuable for peanut butter producers with respect to peanut selection and process optimization aimed at improving product quality.

## 2. Peanut Varieties and Cultivars

**Figure 1:**
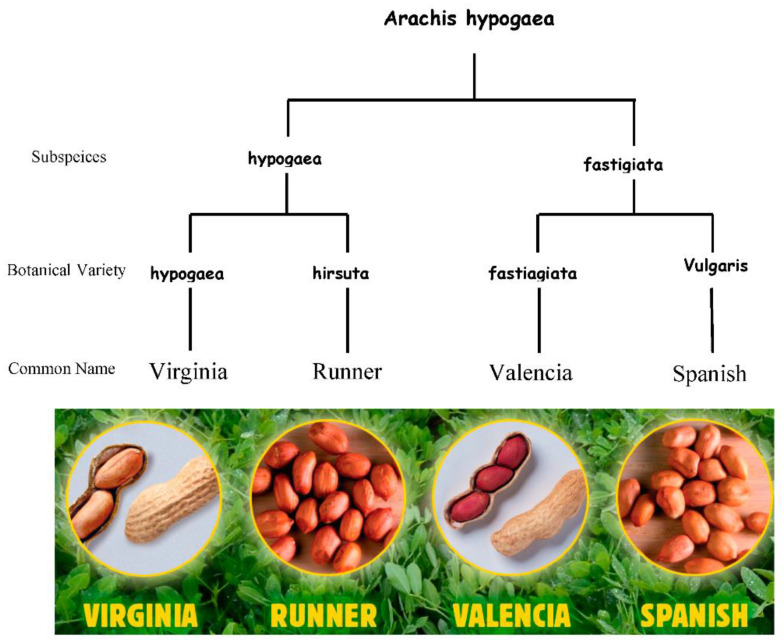
Image showing taxonomic arrangement of the four main subspecies of peanuts in the United States of America.

Peanuts (Arachis *hypogaea*) are the major ingredient used to manufacture peanut butter. The cultivated species of peanuts (Arachis *hypogaea* L.) is an allotetraploid (genome AABB) and comes from the *Arachis* segment of the *Luguminosae* family [29,30,31]. Arachis *duranensis* and Arachis *ipaënsis*, having gone through a series of multiple hybridizations and duplication processes, are believed to be the two progenitors of the modern cultivated peanut varieties [30,32]. Further, the cultivated peanuts (Arachis *hypogaea* L.) can be classified into subspecies: subsp. fastigiata Waldron and subsp. hypogaea Krap [33]. Subsp. fastigiata contains four botanical varieties: var. vulgaris, var. fastigiata, var. peruviana, and var. aequatoriana; subsp. hypogaea contains two varieties: var. hypogaea and var. hirsuta [29,31]. The taxonomic arrangement of the four main subspecies of peanuts in the United States of America [34,35] is shown in Figure 1.

The characteristics and uses of some of the most popular peanut varieties has been detailed by many authors [34,36,37,38,39,40,41]. Table 1 shows some of the most popular peanut varieties, their general characteristics, and their uses.

Over the years, a considerable amount of research has been directed toward the improvement of some of the desirable traits in peanut breeds [31,42]. These peanut breed improvement programs differ widely in scope, with the top priorities evolving around improving yields, improving resistance/tolerance to biotic and abiotic stress factors, developing aflatoxin-resistant breeds, improving the organoleptic qualities of the peanuts, developing reductions in allergenicity, and improving oil quality and quantity [33,43,44,45]. Great advances have been reported in the screening of early generation populations and marker-assisted backcrossing breeding programs, shifting from the conventional use of morphological markers to molecular markers [46,47]. In addition, to complement conventional breeding programs, significant advances have been made in the utilization of genomic tools for high-resolution trait mapping using germplasm diversity panels and data from multi-parent genetic populations [48]. The use of genomic tools has greatly increased the precision and speed at which new peanut breeds with targeted desirable traits are developed and brought onto the market, while circumventing the confounding effects of environmental conditions that are normally encountered in conventional breeding [33,41,48]. Using these recent technological advances, several research studies have been carried out with the aim of amplifying the expression of some desirable traits in peanuts [30,41,49,50,51]. The results of these efforts and many others have brought about considerable variability in the morphological, physiological, and agronomic traits of cultivated peanuts [1,29,52,53,54], thereby inadvertently altering the chemical composition and metabolite profiles of peanuts in a significant way and greatly affecting the organoleptic quality of peanut-based products [55,56]. The variations in marketed peanut kernels include variations in color, size, chemical composition, and nutritional composition and bring about a wider variation in the technical suitability of specific cultivars for particular functions [22]. For instance, regarding kernel color alone, up to 19 peanut kennel colors have been identified, spanning the whole color spectrum from white to very dark purple/blackish [57]. While the parameters shown in Table 2 can be regarded as general average reference values of the chemical composition of peanuts [58], significant variations in these chemical components of peanut cultivars have been reported.

The quantitative and qualitative variations in the composition of peanut metabolomes result from both genetics (cultivar) and growth conditions (individual seeds within an aliquot, year -o-year climate variations, abnormalities of the seeds due to diseases, insect infestations, and the portion of the seeds analyzed); they can provide an essential link that connects peanut genotypes and phenotypes [59]. The actual extent and significance of the variation in the chemical compositions and properties of peanut cultivars have been investigated by several researchers [60,61,62,63,64,65]. With such significant reported differences in the characteristics of cultivated peanuts, there are also expected differences in the sensory quality of peanut butter made from these differing cultivars. The resultant peanut butter organoleptic quality variations brought about by these differences in chemical composition have been found to be perceivable and significant [19,54,66,67,68]. Notwithstanding all such great advances, at the moment it appears that there is limited literature detailing the research of breeding programs that are specifically dedicated to optimizing some peanut breeds for high-quality peanut butter production [69]. In the absence of such peanut breeding programs that are specifically optimized for peanut butter production, peanut butter processors are faced with the burden of sifting through the numerous cultivars that were developed for purposes other than making peanut butter and selecting ones that are most suitable to their objectives and processing conditions. Alternatively, they have to make up for quality inconsistences by continually adjusting their processing conditions to compensate for the variations that are brought about by processing different peanut cultivars.

**Figure 2:**
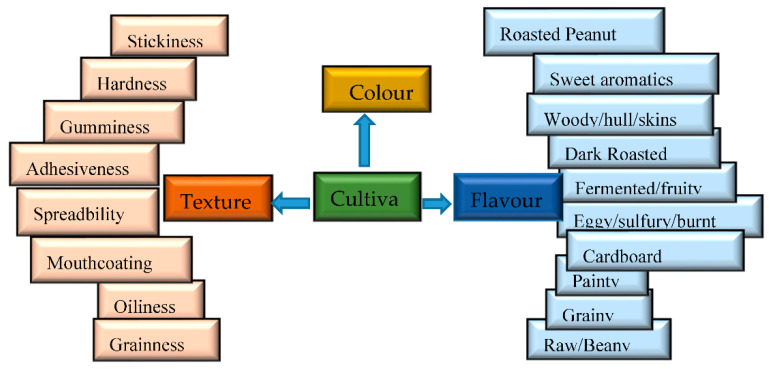
Image showing four of the main quality attributes of peanut butter that are strongly influenced by peanut variety, with some of the applicable lexicon.

The main sensory quality attributes of peanut butter that are affected by variations in the chemical composition and metabolite files of peanuts are flavor, color, and texture. Figure 2 is a pictorial illustration of the main sensory quality indicators that are strongly influenced by variations in the chemical composition of different peanut cultivars, accompanied by the respective lexicon descriptions [9].

## 3. Influence of Peanut Variety on Peanut Butter Flavor

**Figure 3:**
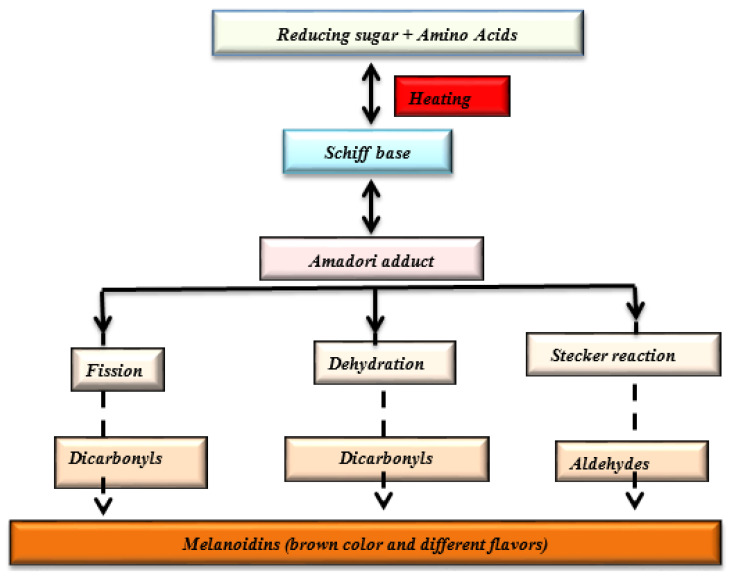
Schematic showing how the Maillard reaction results in color and flavor generation. The reduction of sugars and amino acids in peanuts reacting at high temperatures with low water activity during roasting generates a brown color and various flavors.

For peanut butter, flavor is one of the most important quality attributes, with strong influence on consumer acceptance [70]. Human interpretation of the notes of peanut butter flavor involves the combined experience of the gustatory, olfactory, and trigeminal systems [71]. The olfactory system processes aroma (smell/odor) which is mostly linked to low-molecular-weight volatile compounds of peanut butter. The gustatory system is involved with the taste (sweet, bitter, salty, sour, and umami) sensation that is stimulated by mastication of the non-volatile high-molecular-weight components. Somatosensory perception (astringency, pungency, and acridness) is interpreted by the trigeminal nerves [71,72]. Peanut butter flavor is generated during roasting, when the high temperatures of the roasting process initiate a series physical changes and chemical reactions involving the peanuts’ chemical constituents and metabolites. The reactions to these high temperatures can significantly alter the peanuts’ flavor profile [55]. While the actual temperature-time profile of the roasting process is very significant in the development of peanut butter flavor, the specific chemical composition and metabolite profile, which provide the precursors to the distinct flavor notes that are generated during roasting, are of equal if not greater importance [7,73]. Proteins, sugars, and lipids are the major precursors of volatile compounds in peanuts, and different kinds of sugars and protein mixtures react differently, resulting in different volatile formations [74]. The principal reaction, believed to be mainly responsible for flavor development during peanut roasting, is Maillard and Strecker degradation [74,75]. This reaction proceeds with the linking of free or bound amino acids, mostly lysine and arginine, to the carbonyl group of simple sugars by nonenzymatic glycosylation to form Amadori products on the proteins [76,77]. The Amadori products then undergo rearrangements, cyclizations, and dehydrations to form structurally diverse compounds that are known as advanced glycation end (AGE) products [76]. The resulting structures, mostly pyrazines, pyrroles, pyridines, carbonyl and sulfur compounds, are responsible for the development of the roasted peanut flavor [74,75]. In general, the pyrazines and carbonyl compounds contribute to the desired peanut butter flavor [78] while, on the other hand, sulfur-containing compounds (specifically methionine and cysteine) are primarily responsible for the burnt notes in peanut flavor [75]. Development of color and flavor during roasting involves a number of stages [79]. The stages in the development of flavor and color in peanuts during roasting are illustrated in Figure 3.

While significant efforts have been made to identify and profile the potent odorants in peanuts [80,81,82], the specific compound(s) or groups of compounds that are precursors, and the actual reaction pathways and mechanisms responsible for the typical roasted peanut flavor, remain elusive [71,83]. A clear understanding of the roles of the specific chemical constituents and metabolites of peanuts that are precursors to aroma-active compounds; the biosynthetic pathways for volatile flavor compounds; and the genes that regulate the synthesis of these precursor compounds, is of key importance in improving peanut butter flavor [84].

In 1967, work by Newell et al. [85] demonstrated that off-flavors were highly associated with the presence of threonine, tyrosine, lysine, and an unknown amino acid, while aspartic acid, asparagine- glutamine, glutamic acid, phenylalanine, and histidine were judged to be essential in the development of desirable peanut flavors. In Maillard reaction models, α-amino carbonyl or α-amino hydroxy compounds are believed to be the precursors of pyrazines, and higher concentrations of glutamine, asparagine, diglycine, and triglycine are expected to result in the generation of more pyrazines and, subsequently, a typical roasted/nutty flavor [74]. Significant variations in the amino-acid composition of different peanut varieties have been reported [86]. In 1967, Newell et al. [85] emphasized that the degradation of free amino acids during roasting had a positive correlation to the original concentration. A positive correlation between total sugars and the roasted peanuts’ attributes and a negative correlation between total sugars and bitterness/astringency have also been reported by Pattee et al. in 2000 [87]. It has also been established that the sweetness trait, which is a function of the carbohydrates content of a cultivar, is a heritable trait [87]. Thus, given any two varieties of peanut market-types, the total carbohydrate assays can be used as a basis for the screening and efficient selection of peanut varieties that have higher chances of producing peanut butter with greater sweetness and a superior roasted flavor [87]. With different peanut cultivars having different initial compositions of sugars and free amino acids, appreciable differences in flavor can be observed during roasting, even under the same conditions of roasting [83]. This probably explains why the Runner type peanut, when roasted under the same conditions as the Virginia type, is generally slower in developing a roasted flavor compared with that of the Virginia type; therefore, the Runner type requires a more aggressive time–temperature profile to produce an equivalent roasted flavor [7]. In 2003, Baker et al. [83] emphasized that even if peanuts from different cultivars were to be roasted to the same final color, some appreciable differences in their flavors could still be observed. In 1982, Pattee et al. [88] observed that the larger-sized Virginia peanut seeds (7.14 mm), which had superior flavor in comparison to small-sized peanut seeds (5.14 mm), had a comparatively lower average concentration of free amino acids (32.3%) related to typical roasted flavor (aspartic acid, glutamic acid, histidine, phenylalanine, and histidine) and a lower concentration of amino acids (59.8%) related to atypical roasted flavor (threonine, tyrosine, lysine, and arginine). This further demonstrated the significant effects of varying the chemical composition of peanuts on the development of flavor, even with the same peanut variety.

### 3.1. Effects of Variation of Lipids Composition on Peanut Butter Flavor

Lipids also have a profound effect on the flavor of peanut butter, contributing to both undesirable and desirable flavors. Discussion on lipids’ contribution to peanut butter flavor usually centers on lipid oxidation and the generation of undesirable flavor notes. However, given that almost half of the weight of peanut butter is lipids, the influence of lipids on taste during mastication, and the possibility generating lipoxygenase-derived lipid-based volatile compounds that are responsible for desirable flavors in peanuts, lipids might require further consideration [72]. Twelve different fatty acids have been reported in peanut oil; of these twelve, only oleic, linoleic, and palmitic fatty acids exist in concentrations above 5%. [89]. These three fatty acids (oleic, linoleic, and palmitic) combined have been reported to constitute about 90% of total peanut oil [90]. Peanuts with oleic content above 80% are generally referred to as high oleic, while standard peanut varieties normally have, on average, 50% oleic fatty acid and approximately 25% linoleic fatty acid [23,56,89,90]. 

The difference in roasted flavor between the high-oleic and normal standard cultivars has been investigated by several researchers; however, this is another area where there is an apparent lack of general consensus among researchers. In 2009, Grosso et al. [91] reported that they found no significant difference in consumer preference between Granoleico (GO-P), a high-oleic cultivar, and Tegua (T-P), a normal oleic cultivar, both from Argentina. In a uniform peanut performance test (UPPT) performed in 2015 by Isleib et al. [92], the mean of a total of 27 high-oleic cultivars were compared to the mean of a total of 32 normal oleic cultivars; no observable variation in sensory attribute intensity, other than stale/cardboard, was reported. In a 2006 study, Isleib et al. [93] showed that high-oleic cultivars exhibited a slightly greater intensity of astringent over-roast in roasted peanuts, together with nutty attributes. However, a principal component and cluster analysis (PCC) on some Argentinian high-oleic peanut genotypes (4896-11-C and 9399-10) and some normal oleic varieties showed that high-oleic varieties received higher consumer acceptance ratings [28]. In addition, in work by Wang et al. in 2016 [94], the overall liking, liking of flavor, sweetness, and roasted peanut flavor of normal-oleic runner cultivar GA 06G were compared with those of the high-oleic GA 13M variety; the results showed that the high-oleic variety ranked higher in all of these attributes, as well as in general consumer preference. 

On the other hand, Hu et al. [74] observed in 2021 that the initial concentration of the characteristic precursors of strong peanut flavor were higher in normal-oleic peanuts than in high-oleic peanuts, resulting in the formation of more typical volatile components and a stronger, specific aroma, even when processed under the same conditions. Further, it is not clear whether the aldehydes formed by lipid oxidation or Maillard reactions (in the form of Strecker aldehydes) act in the same way or otherwise, in tandem, during flavor and color formation [83]. In the assumption that they do, Baker et al. [83] suggested in 2003 that this could mean that high-oleic peanuts have less potential for forming precursors to color and flavor via Maillard browning and subsequent pyrazine formation. Therefore, in comparison to normal-oleic lines, a more intense roasting temperature–time profile will be required for the high-oleic peanuts to achieve comparable flavors and colors [83]. 

While further research is required to reconcile these apparent contradictions and to understand the peanut butter flavor phenomena as a function of fatty acid chemistry, at this time the lack of agreement between researchers probably serves as proof that, indeed, different varieties of peanuts result in significantly different flavor profiles. Only with extensive and large-scale research that incorporates as many different representative cultivars as possible will bring to rest these apparent contradictions on the effects of high-oleic content on the development of desirable flavors during roasting. In any case, as desirable flavor development depends not only on lipid content, but also on other peanut constituents, it will not be surprising if only cultivar-specific generalizations are meaningful.

### 3.2. Effects of Lipid Composition of Peanuts on Oxidation Stability

Lipid oxidation reactions make a significant contribution to peanuts butter flavor [55]. Lipid oxidation affects the flavor of peanut butter in two ways. First, during roasting, the lipids in peanuts can undergo degradation, producing some volatile compounds that can directly alter the flavor profile of peanut butter or indirectly influence the flavor of peanut butter by possibly interacting with other constituents developed from the Maillard reaction and Strecker degradation [72]. Second, during storage, lipid oxidation can lead to a fading out of the desirable peanut butter flavor with time. In both cases, lipid degradation in peanut butter could be initiated by autoxidation, photooxidation, or the presence of metallic ions and, in some cases, a combination of all three. Oxidation proceeds with the generation of aliphatic aldehydes, ketones, and alcohols that are highly associated with undesirable flavors, and which can compromises the nutritional quality of fats, possibly leading to the production of toxic compounds [20,72,80]. Both the total lipid content and the actual oil profile have a effects on the propensity and actual rate of peanut butter deterioration by oxidation and, subsequently, on flavor [20,95]. The percentage distribution between the major peanut oil fractions, oleic and linoleic, is one key determinant of the oxidation stability of peanut butter. Linoleic fatty acid is less saturated and suffers more from oxidation, compared with oleic and palmitic fatty acids [90]. 

Accordingly, in peanuts and their products, the ratio of oleic to linoleic fatty acids (O/L) is used as a quality score; the higher the ratio, the greater the product’s shelf life, due to its higher oxidative stability [96]. The peroxide value (PV) is a widely used indicator of storage stability, due to its superior correlation with peanut butter stability, compared with other measures [54]. In 2018, Gong et al. [54] observed a significant (<0.05) positive correlation between the oleic/linoleic ratio in peanut butter stability, as inferred from the peroxide values, further demonstrating the significance of oleic-acid and linoleic-acid content in peanut butter stability. In 2016, Davis et al. [97] established that the oxidative stability Index (OSI) of peanut butter increased more than sevenfold when the O/L ratio increased from 1.3 to 33.8, and the obtained response fit well in a second-order polynomial relationship. 

Wide variability in O/L ratios among peanut cultivars has been reported. In 1987, within some experimental breeding lines in Florida, Norden et al. [98] reported a high variability in the oleic/linoleic ratios, ranging from 0.9 to as high as 35. In Pakistan, peanut cultivars Bard-479 and Local-334, with high oleic/linoleic ratios, were judged to produce better peanut butter in comparison to Bard-92, which has a low oleic/linoleic ratio [69]. Of the three, Bard -479, a Virginia large-seeded cultivar, was recommended for making peanut butter, due to its comparatively high O/L ratio [69]. Among the natural breading lines, the Spanish and the Valencia are generally known to have lower O/L ratios and, subsequently, lower oxidation stabilities, in comparison to the Virginia or Runner types [39,89]. However, in the wake of several breed improvement programs, these generalizations are diminishing. For example, it has been reported that a Runner peanut cultivar, IAC Runner 886, is highly susceptible to oxidation, due to its high percentage of unsaturated fatty acids [96]. In 2021, Huang et al. [52] considered the lipidomic characteristics and free fatty acids of 13 peanut cultivars and compared their lipid compositions. They obtained a significant difference in lipid composition and oxidation stabilities, even among the high-oleic-acid (OA) peanut cultivars [52]. Moreover, they obtained 11 lipid molecules with the potential to be used as indicators for identifying high-OA and non-high-OA peanut cultivars. Thus, these lipid molecules can be used for the screening and determination of the suitability of a given peanut variety for a given function.

Although the total oil content and the lipid profile have profound effects on the stability of peanut oil, the effects of variability in moisture, tocopherols, beta carotene, and chlorophyll should also be considered [20]. The effects of these functional compounds in peanut oil have long been studied and established; thus, they might = be applicable to peanut butter.

### 3.3. Variations in Mineral Constituent of Peanut Cultivar and Oxidative Stability

The mineral constituencies of peanuts also vary with peanut varieties. In 2019, Shibli et al. [69] observed a significant variation in the P, K, and Na mineral constituents of three indigenous Pakistan peanut varieties. It has long been established that metalloproteins, iron, and copper salts are major catalysts of fatty acid oxidation in peanut butter [99]. Further, it is known that contamination of peanut oil with some metallic elements from an external source can accelerate the oxidation of oils. While, in general, the effect of Cu could be more pronounced than that of Fe, the overall effects of these metals on oxidative stability is less pronounced, compared with that of the oleic/linoleic ratio [97]. In the absence of specific research that focuses on the effects of these metallic elements in peanut butter that is prepared from widely varying peanut cultivars, it remains unclear whether such reported variations in mineral content with cultivars can also result in significant oxidation differences in peanut butter. Perhaps a specific study to ascertain this hypothesis might be worthwhile.

### 3.4. Varieties of Peanuts on Flavor Loss during Peanut Butter Storage

The most significant effects of lipid oxidation on peanut butter flavor are related to losses in flavor due to storage, which is also influenced by the lipid profile of a given peanut cultivar [55]. Insuring that the desirable peanut butter flavor developed during roasting does not quickly fade with time is one of the challenges of peanut butter producers [55,100,101]. The oxidation of the lipid component of peanut butter is the chief cause of loss of flavor in peanut butter [97,102]. However, the actual mechanism of flavor loss in peanut butter is still a contentious issue. In 1996, Warner et al. [101] determined that the deterioration of the peanut flavor with time is not necessarily driven by the absence or fading of essential volatile compounds (pyrazines) for peanut flavors, but rather that it results from the masking effect of these flavors by the process of oxidation. In contrast, Bett and Boylston [103] noted in 1992 that alkylpyrazines and peanut flavor intensities actually decreased in intensity, while the lipid oxidation flavors, such as the painty and cardboard flavors, increased with hexanal, octanal, and 2-octanone compounds. Another possibility for flavor fading that still remains unclear is whether or not the desirable peanut flavor compounds undergo a chemical reaction with the off-flavor compounds during storage time, resulting in new products with undesirable off-flavors. The current general understanding is that when the lipids in the peanuts are oxidized, they produce hydroperoxides, which then further degrade to alcohols, alkanes, ketones, and aldehydes, contributing to the off-flavors in peanut butter with an increase in storage time [102]. The off-flavor development in peanuts generally proceeds in a successive order, starting with a cardboard-like flavor followed by a fish-like flavor, and then a paint-like flavor [55]. Because the secondary off-flavor compounds, such as octanal and nonanal, are dependent on the relative starting composition of the fatty acids, it can be thus concluded that peanut variety can contribute to the rate of flavor loss in peanut butter [55]. High-oleic cultivars have better oxidation stability and, subsequently, increased shelf life, compared with that of conventional cultivars [54,97,104]. When the sensory and oxidation stability of a high-oleic peanut variety (i.e., Granoleico, GO-P) was compared with that of a normal-oleic peanut variety (i.e., Tegua, T-P), both prepared under the same conditions and stored at 4 °C, 23 °C, and 40 °C, the peanut paste prepared with high-oleic peanuts had four (at 4 °C), two (at 23 °C), and three (at 40 °C) times longer shelf-life than peanut paste prepared with the normal-oleic variety [105]. The advantages of high-oleic varieties in conferring a longer shelf life has prompted some studies to use advanced techniques, such as near-infrared reflectance spectroscopy (NIRS) for the rapid identification of peanut varieties with high-oleic composition for the purposes of screening and selecting peanuts [106,107]. Further development and the uptake of high-oleic varieties in mainstream peanut butter production processes could be very advantageous, especially for natural peanut butter formulations that are more prone to oxidation, due to the absence of stabilizers [54].

## 4. Varieties of Peanuts and the Final Color of Peanut Butter 

By far, one of the most important attributes of food that determine initial consumer acceptance and preference, or outright rejection of a food product, is color [11,108,109]. Color generates prior expectations that go a long way in influencing the perception with respect to other attributes, such as taste and flavor [11,110,111]. Numerous experiments have proven that beyond perception, the color of food can also significantly influence the actual orthonasal and retronasal olfaction experiences [11,15,110]. With peanut butter, if the color expectation is not met, it becomes difficult for consumers to imagine that the other quality attributes are in place. The desirable “medium brown” color of peanuts is developed during roasting via the Maillard reaction and the caramelization reaction that mainly involve proteins and sugars [100]. The main proteins in peanuts are albumins and two globulins (arachin and conarachin), while the carbohydrates portion includes starch, pectin, cellulose, and sugars [71]. Six sugars have been identified in roasted peanuts, with approximately 88% of the total sugar content being sucrose, 9% stachyose, and raffinose, glucose, fructose, and myo-inositol constituting the remaining percentage [112]. 

When roasting peanuts, sucrose is hydrolyzed into fructose and glucose by invertase, and then these reducing sugars take part in browning reactions that result in the desirable peanut color [71]. It was observed that during roasting, sucrose concentrations decrease with the increase in roast color [112]. If the roasting process is held as a constant, color development in peanut butter is a function of the constituent components of the peanut cultivar. In 2016, Lykomitros et al. [80] concluded that the color-flavor– relationship depends mostly on the chemical composition of the material, and further cautioned against strong reliance on color as a measure of the extent of roasting, especially in operations that processed different varieties of peanuts. Variations in the protein and sugar content of the peanut cultivar result in expected variations in the color of roasted peanuts and the subsequent level of acceptance of peanut butter [88]. 

In a study carried out in 2018 by Gong et al. [54] on 17 Chinese varieties of peanuts, in some cases the sugar content was found to vary by up to five times. This agreed well with the results obtained in 2019 by Shibli et al. [69] on indigenous Pakistan cultivars. Such variations in the sugar content of peanuts among different peanut cultivars contribute to the color difference in peanuts that are roasted under the same conditions [88]. In one experiment, a significantly higher sucrose content was found in a 5.95 mm size Virginia peanut, compared with another with a 7.14 mm size [88]. The high sucrose content was interpreted as instrumental in the formation of a dark roast color in the smaller-sized peanut seed (5.95 mm) via a caramelization reaction [88]. In their 2016 study on the kinetics of color development during roasting, Shi et al. [113] emphatically concluded (and also cautioned) that the activation energies they had obtained were only applicable to the variety they had used in their experiment. They conceded that even slight differences in chemical composition, such as sugar content, can cause substantial deviations from the desired color. In their respective studies with specific peanut varieties, Dhamsaniya and Patel [23] in 2013 and Shibli et al. in 2019 [69] could not obtain statistical significant results on the color differences of peanut butter produced from different peanut cultivars; however, in both studies, the results from their respective sensory panelist showed significant differences in preference based on color and aroma. In comparison, some high- or mid-oleic genotypes generally require longer roasting times to reach roast colors that are comparable to normal oleic genotypes [83].

### Effects of Variations in Peanut Cultivars on Blanchability and Peanut Butter Color

Blanchability, a measure of capability of a given peanut genotype to recover whole kernels when all the testa is removed, also influences the color consistency of peanut butter [114]. Poor blanchability might results in an ingress of peanut skins into peanut butter, compromising color consistency. Blanchability is a heritable trait; therefore, breeders can incorporate this attribute in their breading programs to improve the overall marketability of their peanut varieties [114,115]. Furthermore, peanut oil content has a positive association with blanchability, while protein content and kernel length have a negative correlation with blanchability [114]. On the other hand, peanut kernel size can also affect the color of peanut butter; if peanuts of different kernel sizes are roasted under the same conditions, the larger kernel size (due to its small relative surface size per unit mass) will be slow in developing color when compared to smaller-sized kernels. This demonstrates the complex interdependence between peanut kernel characteristics, peanut chemical constituents, and their effects on sensory quality attributes. Therefore, in addition to the optimization of the roasting process for good color development, it is imperative that only peanut cultivars known for superior color development are selected and processed.

## 5. Effects of Variation in Peanut Varieties on the Textural Properties of Peanut Butter

In addition to flavor and color, the texture of peanut butter is a very important attribute that determines eating quality and consumer satisfaction [116]. The mastication quality of peanut butter can either generate memorable enjoyment, which can motivate repeated consumption, or lead to total abhorrence [117]. If peanut butter is too sticky, it results in poor spreadibility and adheres to the teeth, tongue, gums, and palate, resulting in great difficulties in swallowing, causing discomfort and anxiety, and ultimately compromising consumer enjoyment [117]. On the other hand, if peanut butter is too oily, it can be runny and difficult to spread, and it can easily cause a mess. The texture of peanut butter is to a great extent a function of grinding and stabilization processes [21]. However, some variations in textural and rheological properties of peanut butter that have undergone the same grinding and stabilization processes have been attributed to differences in the chemical composition and metabolite profiles of the peanut cultivars. Different peanut varieties can produce peanut butter with significantly different textural properties, even when processed under identical conditions.

Some studies have shown that sucrose has a significant positive correlation with the firmness, cohesiveness, and the rheology (yield stress, K, G′ -a, and G′′-c) of peanut butter [22]. Higher sucrose levels can possibly result in increased macromolecular entanglement and subsequent improvements in firmness and cohesiveness [22]. Peanut proteins, which constitute the bulk of the solid phase of the peanut butter matrix, also have a significant effect on the textural and rheological properties of peanut butter. Arachin (14s) and conarachin (8S, 2S) are the major proteins found in peanuts and peanut butter, with arachin constituting about 63% of the total peanut seed proteins [118,119,120]. As a results of genetic and environmental variations, different peanut varieties may have significantly different arachin and conarachin contents [118]. The conarachin content is positively correlated with hardness, springiness, and cohesiveness of peanut gel, while the arachin content and the arachin/conarachin ratio are negatively correlated with the gel’s textural properties [119]. Discrepancies in the subunit composition of these arachin proteins have a profound influence on their functional properties [119,120]. It has been reported that some peanut varieties do not have the 35.5 kDa subunit in their arachin protein structure [120]. Arachin’s protein structure, containing the 35.5 kDa subunit, has higher surface hydrophobicity, which is probably due to the relatively higher proportion of hydrophobic amino acids on the surface, while those without have more disulfide bonds on the surface and, therefore, have a more structurally and thermally stable globular matrix [120]. These differences are likely to influence how the proteins will interact with the lipids, thereby determining the rate of oil separation and, ultimately, the texture and rheological properties of peanut butter. Proline (Pro) and cysteine (Cys) were observed to be significantly and positively correlated with the texture (firmness and cohesiveness) and rheological (yield stress) characteristics [22]. The relatively higher hydrophobicity of proline can possibly increase the stability of the protein space structure, while cysteine is likely to increase stability in the protein matrix by forming disulfide bonds. Sugars are believed to decrease the thermal unfolding of arachin, which strengthens the intra-protein hydrophobic and hydrogen bonding [120]. In general, total lipid content has a negative relationship with the texture and rheological characteristics, such as firmness. This is mainly because the continuous lipids phase acts as a lubricant in the peanut butter matrix, and its reduction is likely to increase the viscosity of the peanut [22]. 

Several studies ascertained the significance of the effects on peanut butter texture and rheological properties. Due to variations in peanut varieties Dhamsaniya and Patel [23], in their 2013 study on peanut butter made from seven different peanut cultivars in India, observed significant differences in adhesiveness and spreadability. In a similar study in 2014, Rozalli et al. [121] also reported some differences in textural properties between peanut butter made from Chinese varieties and peanut butter made from Indian varieties. Although consumers in Argentina could not identify significant appreciable differences between peanut butter made from high-oleic and normal-oleic cultivars from Argentina, they did note the textural differences between those two cultivars in terms of oiliness [122]. In a study carried out in 1986 by Ahmed and Ali [122] on peanut butter made from Florunner seeds (50.1% oil), pressed Florunner seeds (39.9% oil), and Jamaican seeds (39.9% oil), some significant variations in textural properties, most probably as a result of the differences in oil content, were observed. Significant differences in density and viscosity have been reported between high- and normal-oleic peanut cultivars [56]. In comparison with low-oleic cultivars, high-oleic cultivars were observed to have low densities and high viscosities [56,97]. Because peanut butter’s textural properties are also influenced by the mobility of oil droplets within the peanut’s solid phase matrix, the differences in the density and viscosity of peanut oil would affect the peanut butter texture and could possibly further influence the stabilization requirements of peanut butter. 

Based on the forgoing analysis, it is clear that the differences in the chemical composition and metabolite profiles of peanuts of different peanut cultivars have significant effects on the textural properties of peanut butter. Thus, different peanut varieties will produce different types of peanut butters of significantly different eating, with different consumer acceptance ratings [69,123].

While this paper has attempted to account for and explain the variations in the sensory qualities of peanut butter that emanate from variations in the chemical composition and metabolites of peanuts, it is evident that the interactions that govern these relationships are fairly complex. For example, increasing the O/L ratio will significantly improve the oxidation stability of peanut butter, while at the same time significantly altering the textural and rheological properties. Again, breed improvement programs that target better flavor in peanut butter by increasing the sucrose content of peanuts are likely to result in cultivars with lower oil content and high protein content [22,123]. Furthermore, although the target might be altering the chemical composition and metabolite profile for superior organoleptic properties, some physiological changes can be inadvertently introduced, such as changes in kernel size, which also affect roasting characteristics and, subsequently, color, flavor, and texture. The knowledge gaps that are highlighted in this paper show that a comprehensive study on peanut breeding lines that specifically details how variations in chemical composition affect the major and most significant quality attributes of peanut butter, and the interrelations between these attributes, is still needed.

## 6. Conclusions

Apart from processing conditions, the specific chemical composition and metabolite profile of a peanut cultivar has a very strong influence on the organoleptic properties of peanut butter and, subsequently, on peanut butter’s acceptance by consumers. Rapid advances in the science of peanut breading are resulting in quick delivery of new peanut varieties to markets albeit with widely differing chemical compositions and metabolite profiles, which in turn exhibit a widely varying degrees of aptness for making peanut butter. Given that, in most cases, the primary focus of these breeding programs has little or no connection with optimum peanut butter production, it can be a challenge for peanut butter producers to consistently source peanuts with inherently superior organoleptic qualities from the open market. Based on this article’s discussion, it is clear that differences in the chemical composition of peanuts are very significant, with significant effects on the sensory quality of peanut butter. Therefore, food scientists need to unpack the relationships between desirable sensory qualities in peanut butter and the respective metabolite precursors to those qualities, as well as the relevant interdependences among these precursors. Furthermore, genetic mapping can be carried out to identify genes and biological processes that underlie the production of desirable traits that are influenced by inheritance, thereby providing a solid base for improving peanut breeding programs that are specific and optimum for peanut butter production. A breakthrough in that manner might lead to manufacturers making significant improvements in the quality of peanut butter, as they will be able to select peanut breeds that have been specifically optimized for peanut butter production.

## Figures and Tables

**Table 1:** Characteristics and uses of the most popular peanut varieties.

	*Popular Varieties in US*	*Characteristics*	*Popular Uses*
**Virginia**	Bailey, Champs, Florida Fancy, Gregory, Perry, Phillips, Sugg, Sullivan, Titan, and Wynne	Large-sized kernel, generally elongated, and tapered towards the sprout end. Pinkish-tan skins when fresh, which change to reddish-brown color with storage. Skin texture is comparable to the Runner variety.	Snacking cocktails and partial use in peanut butter.
***Runner***	Florunner, Sunrunner, Southern Runner, Georgia Runner, Georgia Green, and Flavor Runner 458	Medium-sized, uniformly shaped kernels that are elongated with blunt flattened ends. Rougher skins in comparison to the Spanish type. Color is generally pinkish-brown when fresh and turns to reddish brown with storage. Large yield output and medium-sized and uniformly shaped kennels. Relative uniformity during roasting.	Peanut butter production.
***Valencia***	Tennessee Reds	Cylindrical and slender pods, containing two to four kernels with blocky, flattened ends. Kernels are small to medium in size with a distinctive bright red color when fresh, turning dark red with storage. A distinctive sweet taste.	Homemade peanut butter and fresh boiled peanuts.
***Spanish***	Georgia-045, Olin, Pronto, Spanco, and Tamspan 90	Distinctive rounded shape and relatively small-sized kernels for older varieties and medium-sized kernels for new varieties. Smooth skin and delicate texture; color changes from pinkish buff when fresh to a light brown with an increase in storage time. Their distinctive reddish-brown color and small size make them most suitable for candies and salted-shell nuts. High oil content and a distinctively strong nutty flavor.	Oil production, candies, and salted shelled nuts.

**Table 2:** Chemical and nutritional composition of peanuts.

*Components*	*Class*	*Types*	*Amount (Per 100 g of Dry Roasted Peanuts)*
***Lipids***	Fatty acids	Saturated	6.893 g
		Monosaturated	24.640 g
		Polysaturated	15.694 g
***Vitamins***	Fat soluble	E (tocopherol)	8.2 mg (raw), 4.1 mg/100 g roasted
	Water soluble	B2 (Riboflavin)	0.098 mg
		B1 (Thiamine)	1.0 mg
		B5 (Panthothenic acid)	1.395 mg
		B3 (Niacin)	13.525 mg
		B6 (Pyridoxine)	0.256 mg
		B9 (Folate)	145 mg
		Choline	55.3 mg
***Minerals***	Macro	Potassium	658 mg
		Sodium	Approx. 5.56 mg
		Calcium	54 mg
		Magnesium	175 mg
		Phosphorus	358 mg
	Micro	* Selenium	7.5 mg
		* Copper	0.671 mg
		* Manganese	Approx. 2.06 mg
		Iron	2.26 mg
		Zinc	3.31 mg
		(* antioxidant minerals)	
***Amino acids***	Essential	Tryptophan	0.230 gm
		Leucine	1.535 gm
		Isoleucine	0.833 gm
		Methioione	0.291 gm
		Phenyalanine	0.304 gm
		Valine	0.993 gm
		Lysine	0.850 gm
		Threonine	0.811 gm
	Non-essential	Glycine	1.427 gm
		Alanine	0.941 gm
		Cysteine	0.304 gm
		Tyrosine	0.963 gm
		Arginine	2.832 gm
		Histidine	0.599 gm
		Aspartic acid	2.888 gm
		Glutamic acid	4.949 gm
		Proline	1.045 gm
		Serine	1.167 gm
***Others***	Total carbohydrates		21.51 gm
	Total sugars		4.18 gm
	Dietary fibers		8.0 gm
***Bioactive Compounds***	Isoflavonoid	Daidzein	49.7 mg
		Genistein	82.6 mg
	Phenolic acids	p-coumaric acid	6.9 mg
	Phytosterols	b-sitosterol	61 mg to 114 mg
	Stilbenes	Resveratrol	0.48 mg to 3.96 mg
		Coenzyme Q10	

The asterisk represent minerals i.e., Selenium, Copper; Manganese are (* antioxidant minerals) as highlited.

## Data Availability

Not applicable.
